# Supplementary material for: Coffee Oil Extraction Methods: A Review
Source: Foods. 2024 Aug 20;13(16):2601. doi: 10.3390/foods13162601 (PMC11353398; doi:10.3390/foods13162601)
Supplement: Supplementary file 1 [file foods-13-02601-s001.zip › foods-3141738-supplementary.pdf]

SUPPLEMENTARY MATERIAL

Table S1. Coffee Oil Extraction Methods

(continue)

Methods	Coffee bean	Process	Conditions described	Oil yields (%)	Reference
**Conventional methods**					
Expeller Press	Arabica	Green and Roasted	2 cycles	10.8 (Healthy green);<br>9.2 – 10.0 (Defective green);<br>10.3 (Healthy roasted);<br>9.0 – 10.3 (Defective roasted)	[68]
	Arabica	Green	40 and 45 °C	4.21	[69]
	Arabica	Green	Particle size (0.850 - 2.00 mm);<br>18 and 30 rpm;<br>press exit nozzle size (4 and 5 mm);<br>preheating (on and off)	2.65 - 6.27	[61]
Soxhlet	N/I	N/I	PE;<br>4h	N/I	[73]
	N/I	N/I	PE;<br>16 h	N/I	[74]
	Arabica and Robusta	Green	HEX;<br>8 h;<br>syphoning 6 times per hour	15 - 20 (Arabica);<br>12 – 17 (Robusta)	[76]
	Arabica	Green and Roasted	Ratio of sample to solvent 1:10 (g/mL);<br>HEX;<br>16 h	11.37 (Green);<br>15.49 (Roasted)	[77]

(continuation)

Methods	Coffee bean	Process	Conditions described	Oil yields (%)	Reference
Soxhlet	Arabica	Green	HEX;<br>16 h	10 – 12 (Healthy and defective)	[78]
	Robusta	Green and Roasted	40 - 60°C;<br>PE;<br>1 h	10.22 (Green);<br>10.15 - 11.31 (Roasted)	[55]
	Arabica	Roasted	Ratio of sample to solvent 1:30 (g/mL);<br>TBME;<br>6 h	18.6	[79]
	Arabica	Green	Ratio of sample to solvent 1:4 (g/mL);<br>PE;<br>4 h	7.49 - 9.47	[81]
	Arabica	Green	Ratio of sample to solvent 1:5 (g/mL);<br>ME2CO, EtOH, EtOAc, HEX, IPA, and PE;<br>3 and 5 h	8.31 – 8.47 (ME2CO);<br>11.78 – 14.16 (EtOH);<br>6.44 – 9.77 (EtOAc);<br>8.31 - 8.85 (HEX);<br>10.23 - 11.21 (IPA);<br>7.67 – 7.94 (PE)	[84]
	Arabica	Green	Ratio of sample to solvent 1:40 (g/mL);<br>HEX;<br>9 h	7.57	[99]
	Arabica	Green	Ratio of sample to solvent 1:7,5 (g/mL);<br>HEX;<br>4 h	9.05 (Healthy);<br>9.47 (Defective)	[85]

(continuation)

Methods	Coffee bean	Process	Conditions described	Oil yields (%)	Reference
Extrusion followed by Soxhlet	Arabica	Green	Extrusion:<br>40 - 80 °C;<br>60 - 100 rpm<br><br>Soxhlet:<br>Ratio of sample to solvent 1:7,5 (g/mL);<br>HEX;<br>4 h	16.42 (Healthy);<br>15.29 (Defective)	[85]
Microwave-assisted extraction	Arabica	Green	Ratio of sample to solvent 1:4 (g/mL);<br>EtOH;<br>30 - 45 ºC;<br>2 - 10 min;<br>600 rpm	5.86 - 7.61	[81]
	Arabica	Green	Ratio of sample to solvent 1:10 (g/mL);<br>EtOH;<br>60 °C;<br>30 min;<br>200 W	9.34	[103]
Supercritical fluid extraction	Arabica	Green and Roasted	CO_2;<br>60 - 90°C;<br>235 - 380 bar;<br>25 min	11.37 (Green);<br>15.49 (Roasted)	[77]
	Arabica	Green	CO_2, CO_2/ EtOH (5%) and CO_2/IPA (5%);<br>50 and 60 °C;<br>152 - 352 bar;	N/I	[97]

(continuation)

Methods	Coffee bean	Process	Conditions described	Oil yields (%)	Reference
Supercritical fluid extraction	Arabica	Green	CO_2; 65.9 - 94.1 °C; 174.5 - 325.5 bar; 20 min	0.01 – 6.50	[100]
	Arabica	Roasted	CO_2; 40 and 60 °C; 150 - 300 bar; 120 min	0.59 – 7.38	[98]
	Arabica	Green	CO_2; 40 - 80 °C; 300 and 350 bar	5.95 – 7.60	[99]
	Arabica	Green	CO_2/ EtOH (0 - 5.7%); 40 - 60 °C; 200 - 400 bar	N/I	[100]
	Arabica	Green	CO_2/ EtOH (5 - 20%); 50 - 70 °C; 15 - 30 bar	0.6 - 8.1	[114]
**Non-conventional methods**					
Hydraulic Press	N/I	Roasted	Room temperature; 350 - 550 bar; 60 min	2.47 (Defective)	[104]
Ultrasonic/microwave-assisted extraction	Arabica	Green	Ratio of sample to solvent 1:28 (g/mL); EtOH; 60 °C; 10 min; 350 W	10.58	[103]

(conclusion)

Methods	Coffee bean	Process	Conditions described	Oil yields (%)	Reference
Ultrasound-assisted extraction	Arabica	Green	Ratio of sample to solvent 1:30 (g/mL); EtOH; 35 °C; 50 min; 40 kHz; 50 W	9.06	[103]
Pressurized liquid extraction	Arabica	Green	Ratio of sample to solvent 1:2 (g/mL); EtOH; 100 °C; 30 min; 100 bar	6.34	[103]

* N/I: not identified.
Solvents: PE: Petroleum ether; HEX: Hexane; EtOH: Ethanol; ME2CO: Acetone; TBME: *t*-butyl methyl ether; EtOAc: Ethyl acetate; IPA: isopropyl alcohol.
